# Aphallia - congenital absence of the penis: a systematic review

**DOI:** 10.1186/s12894-024-01445-4

**Published:** 2024-03-28

**Authors:** Prahara Yuri, Peri Eriad Yunir, Eldo Taufila Putra Utama, Yevri Zulfiqar, Jarir At Thobari

**Affiliations:** 1 Division of Urology, Department of Surgery, Faculty of Medicine, Public Health and Nursing, Universitas Gadjah Mada/Dr. Sardjito Hospital, Jl. Kesehatan No. 1, Yogyakarta, 55281 Indonesia; 2 Clinical Epidemiology and Biostatistic Unit (CEBU) Faculty of Medicine, Public Health and Nursing, Universitas Gadjah Mada/Dr. Sardjito Hospital, Jl. Medika, Yogyakarta, 55281 Indonesia; 3 Division of Urology, Department of Surgery, Faculty of Medicine, Universitas Andalas, M. Djamil Hospital, Padang, Indonesia; 4 Department of Pharmacology and Therapy, Faculty of Medicine, Public Health and Nursing, Universitas Gadjah Mada, Yogyakarta, Special Region of Yogyakarta Indonesia

**Keywords:** Aphallia, Congenital anomaly, Fistula, Vesicostomy, Sex determination

## Abstract

**Background:**

Aphallia is a rare congenital anomaly often associated with other urogenital anomalies. The management of aphallia cases for both the immediate and long-term treatment of patients with aphallia pose a major dilemma. Patients are at risk for psychosocial and psychosexual challenges throughout life.

**Methods:**

A systematic review was conducted on aphallia cases. We searched online databases until March 2023 for relevant articles and performed according to the PRISMA-P guidelines.

**Results:**

Of the 43 articles screened, there were 33 articles included. A total of 41 patients were analyzed qualitatively. Asia is the region with the most aphallia cases with 53% (n:22), while the United States is the country with the most most reported aphallia cases 31% (n:13). Most cases were identified as male sex (n: 40), and most cases were neonate with 68% (n:28) cases. Physical examination generally found 85% (*N* = 35) with normal scrotal development and palpable testes. The most affected system with anomalies is the genitourinary system with fistulas in 80% (n:29) cases. Initial management in 39% (n:16) of patients involved vesicostomy. Further management of 31% (n:13) included phalloplasty or penile reconstruction, and 12% (n:5) chose female sex. 17% (n:7) of patients refused medical treatment or were lost to follow-up, and 12% (*n* = 5) patients deceased.

**Conclusion:**

Aphallia is a rare condition and is often associated with other inherited genitourinary disorders. In most cases, physical examinations are normal except for the absence of a phallus, and laboratory testing shows normal results. The initial management typically involves the vesicostomy procedure. Subsequent management focuses on gender determination. Currently, male sex is preferred over female. Due to the significant variability, the rarity of cases, and the lack of long-term effect reporting in many studies on aphallia, further research is needed to minimize bias.

## Background

Aphallia, is a rare congenital anomaly (1:30 million) and is associated with other urogenital anomalies. Under 100 cases of aphallia have been reported worldwide [1]. The absence or failure of the development of the genital tuberculum will cause aphallia. The penis and clitoris originate from the genital tubercle, which develops from mesenchymal prominence in the cloaca membrane during the fourth week of pregnancy. This failure may be caused by an initial disruption in cloacal maturation or poor development of the caudal mesenchyme. This can also lead to proximal urethrorectal communication, which will explain the high incidence of concurrent anorectal anomalies seen in aphallia patients [2].

Both the immediate and long-term management of aphallia patients pose a major dilemma [1] Patients are at risk for psychosocial and psychosexual challenges throughout life [3]. These patients require a multidisciplinary approach that often includes psychologists, endocrinologists, pediatric urologists, pediatric surgeons, plastic surgeons and social workers because not all community health care centers have access to every one of the specialties mentioned above [3]. In this review we aim to address the variability of disease presentation, the associated anomalies of the disease and will review the current knowledge of the treatment options in all age patients of aphallia.

## Material and methods

### Search strategy

We included case reports/ series involving patients with aphallia. This review follows the guidelines in the Preferred Reporting Items for Systematic Reviews and Meta-Analyses—Protocol (PRISMA-P) to increase comprehensiveness and transparency of reporting [4]. We searched two electronic databases, Medline and Sciencedirect, until March 2023. The following keywords and medical subject headings were used: Aphallia, penile agenesis, absence of penile shaft, absence of penis, clitoral absence, absence of phallus, and absence of corpora cavernosa.

### Study selection

The database search was conducted by two authors (P.Y. and E.T). Eligibility criteria for inclusion in the review was a specific focus on aphallia in all age of patients. Operation technique focus and non-English articles were excluded.

All articles reporting one or more cases of aphallia were obtained in full-text. Two authors (P.Y. and E.T) independently extracted data from all studies into data summary tables. The articles which fulfilled the eligibility criteria were included in this review. The following data were collected: age, clinical findings, supporting investigations, laboratory findings and management. The final selected papers were reviewed by three authors (P.E.Y., Y.Z., J.A.T.)

### Data extraction and synthesis

The information that was extracted included the following: author and year of study; demographics of the patient, including age, proportion of males and female, weight and country, presenting signs and symptoms with percentage distribution; laboratory data and imaging findings with percentage distribution; and management with percentage distribution. Due to the qualitative, summative nature of this review and significant variations in participants, presentation, laboratory data, imaging and treatments, a meta-analysis could not be conducted and effect sizes could not be calculated.

## Results

**Fig. 1:**
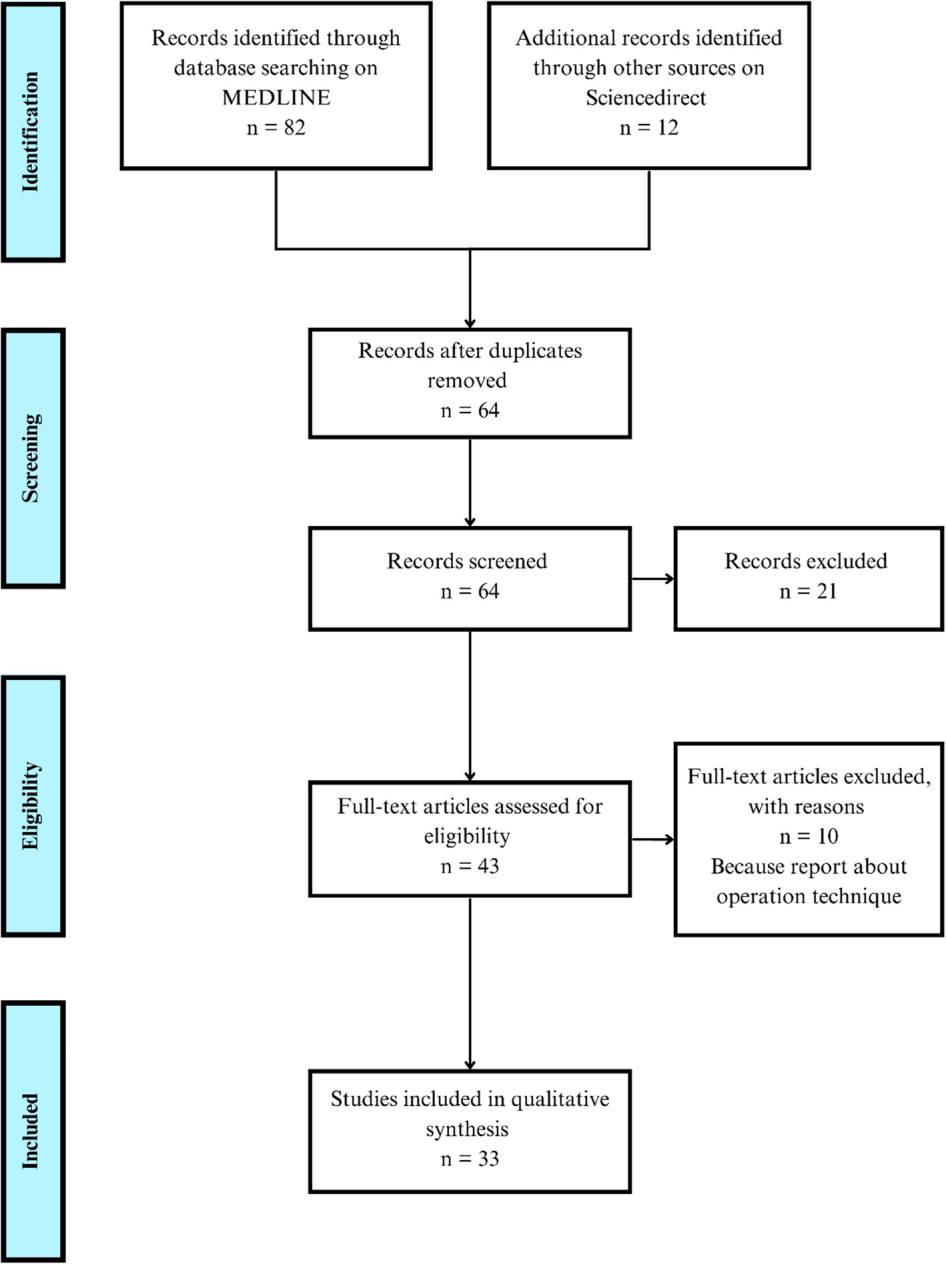
Prisma flow chart

Our search resulted in 94 potentially relevant articles, of which 30 articles are duplicates. From the 64 articles identified, a total of 43 articles were screened for eligibility; and 33 articles met the full inclusion criteria (Fig. 1).

They included 41 patients with aphallia. The results of these 33 studies are summarized in Table 1.

**Table 1:** Study characteristics

Study	Age	Weight	diagnosis	Clinical findings	Supporting investigations	Laboratory findings	Managements
1. Kian et al., 2022 [5]	14 months	2.7 kg	Aphallia	• Penile absent • OUE not detected • Passing urine from anus • Scrotum developed • Bilateral descended testes	• USG: Hipoplasia left kidney, mild diffuse wall thickening of uninary bladder • CT Scan, IV contrast, Hipoplasia of left kidney • Rectal enema with soluble contrast: Vesico rectal fistula, normal anus closure		
2. Luo et al., 2022 [6]	8 years old	-	Absence of penis	• Penile absent • Orificium urethræ externum not detected • Passing urine from anus • Scrotum developed • Bilateral descended testes	• Anterograde urography: Fistula urethrorectal • Echocardiography, electrocardiogram, Chest CT, USG Abdominal normal	• Karyotype chromosome: 46,XY • Blood chemistry and hematology test: Normal limits • Urine analisys: Normal limit	• Urethral exteriorization via perineal urethroplast • Planned nephalloplasty at the time of puberty (age 10–15 years)
3. Braun et al., 2022 [7]	3 years Old	-	“Aphallia, norectal malformation without fistula	• Absent of phallus • Normally descended testes • Anorectal malformation • Perineal urethra	• The renal and bladder ultrasound, CT Scan, IV contrast: Malrotated kidneys • Magnetic resonance imaging: No visualisation of the corpora cavernosa or corpus spongiosum, normal sacrum and spinal cord	-	• Colostomy during the neonatal period followed by posterior-sagittal anorectoplasty • Perineal urethral closure • Suprapubic catheter (SPC) placement
4. Vaizher et al., 2021 [8]	Neonate	-	Aphallia with no associated fistula, and complete urethra atresia”	• Absent of phallus • Scrotal sac with undescended testes • Posterior spinal defect concerning for open neural tube abnormality • Imperforate anus • 2-vessel umbilical cord	• Ultrasound prenatal: Large, multilobulated anechoic umbilical cyst near the fetal cord insertion measuring 5.6 × 3.2x3.0 cm, 2-vessel cord left-sided pyelectasis (renal pelvis measuring 6.1 mm), bilateral hydroureters. Amniotic fluid index (AFI) was normal (17.5 cm) • Ultrasound postnatal: Neural tube defect, atretic left kidney, grade 1 rightsided hydronephrosis • MRI: Distended bladder, grade 3 bilateral hydronephrosis, the bladder terminated into a cystic dilation of proximal urethra without further urethra seen no external nor internal urethral connections • Intraoperative flexible cystoscopy: Revealed minimal trabeculations, no vesicoureteral reflux on cystogram, and an atretic urethral opening at the bladder neck.	• Karyotype chromosome: 46,XY	• Vesicostomy • Sacral laminectomy • Detethering of the spinal cord, dermal sinus tract repair • Descending end colostomy with mucous fistula formation on DOL-1
5. Qadhiry et al., 2020 [9]	Neonate	3.4 kg	Aphallia, urethrorectal fistula	• Penile absent • OUE not detected • Scrotum developed • Bilateral descended testes • Normal anal opening	• USG: Bilateral normal size kidney, bilateral ureterohydronephrosis • Cystourethrography: Grade 3 unilateral vesicoureteral reflux (VUR), urethrorectal fistula	• Karyotype chromosome: 46,XY • Renal function test were normal	• Cystostomy operation
6. Bencharef et al., 2022 [10]	Neonate	-	Aphallia	• Penile absent • OUE not detected • Scrotum developed • Bilateral descended testes • Normal anal opening	• USG: Bilateral hydronephrosis, bilateral hydroureter • Cystography: Urethrorectal fistula Associated with grade 3 left vesicoureteral reflux	• Karyotype chromosome: 46,XY • Plasmatic urea and creatinine tests: Demonstrated renal failure • Hormonal:17-hydroxy progesteron, testosteron, serum dihydrotestosterone, luteinizing hormone (LH), follicule-stimulating hormone (FSH) level within the normal range.	• Vesicostomy • Prophylactic antibiotics to prevent urinary tract infection • Planned Neophalloplasty
7. E Decker et al., 2020 [11]	Neonate	3.3 kg	Abnormal genitalia and anorectal malformation (complete absence of penile shaft)	• Complete absence of penile shaft with well-formed scrotum.	• Ultrasound: Confirmed a solitary left kidney with hydro-uretero-nephrosis.	• Karyotype chromosomes: 46,XY	• Defunction colostomy was performed on day 11 with suprapubic catheter to correct persistent metabolic acidosis
				• Bilaterally descended testes,	• GI contrast: Showed a grossly dilated rectum and recto-bladder neck fistula.	• Serum creatinine: 45 μmol/L	• Fistula closure with interim urinary diversion (vesicostomy).
				• Urine passing through a normally sited ‘funnel anus’.	• MRI: Confirmed absence of corporal bodies and normal pelvic floor musculature.	• Persistent metabolic acidosis secondary to gastrointestinal absorption of urine	• Definitive penile reconstruction is planned during adolescence.
				• Persistent abdominal distension and feed intolerance.			
8. S Qiang et al., 2019 [1]	3 month old	-	Congenital absence of the penis	• Total absence of the penis and urine being passed rectally.	• Ultrasound of the scrotum: Showed the presence of 2 normal testes and epididymis.	• Karyotype chromosomes: 46,XY	• Patient’s parents had not yet decided whether to accept treatment and left hospital without further contact (lost to follow up).
				• The scrotum was well developed, and the testes were palpable bilaterally.	• Instillation of contrast into the rectum and examination by abdominal computed tomography-digital subtraction angiography (CTDSA): Demonstrated a thin tract opening in the rectum, suggesting a urethrorectal fistula.		
				• The anal opening was located normally, and no urethral orifice could be identified.	• Echocardiographic assessment: Found patent ductus arteriosus (PDA), tricuspid regurgitation, pulmonary hypertension, and atrial septal defect (ASD).		
9. J Palacios-Juarez et al., 2018 [3]	1 month old	-	Absence of penis	• Absence of penis and urethra along with a well-formed scrotum and both normally descended testicles.	• CT scan: Revealed a functional but hypoplastic right kidney and the presence of a vesicorectal fistula.	• Hormonal profile were within normal limits.	• The parents only allowing procedure for fistula correction and refused other procedures.
10. R Chaudry et al., 2017 [12]	Neonate0	-	Penile agenesis	• Physical exam at birth was significant for penile agenesis without evidence of a urethral opening.	• 22-week ultrasound: Bilateral renal pelviectasis and hydroureter with no distinct bladder structure, amniotic fluid volumes were normal.	• Karyotype chromosomes: 46,XY	• Initiation of hemodialysis and transitioned to peritoneal dialysis due he patient completely anuric
				• Normal symmetric scrotum with palpable gonads.	• 31 weeks ultrasound: Showed complete anhydramnios		• The parents desired phallic reconstruction. Scrotal skin appeared thin on examination, a decision was made to perform a modified scrotal flap phalloplasty
					• Postnatal ultrasound: Did not identify a bladder or uterus, and the kidneys appeared cystic and dysplastic		
					• Pelvic magnetic resonance imaging at 3 months: Did not definitively reveal a bladder.		
					• At 6 months, patient underwent anoscopy and cystourethroscopy: Anoscopy revealed a small sinus anteriorly at the anal verge, but no evidence of a bladder, the 2 atretic ureters were ectopically inserted into this sinus.		
11. A.A. Friedman et al., 2016 [2]	Case 1: Neonate, Gestational age (GA) 36,4 weeks	-	Penile agenesis and urinary tract dysplasia	• Transient respiratory distress, requiring positive pressure ventilation.	• Renal ultrasound: A small right kidney with parenchymal thinning and partial duplication. The left kidney was normal in size and echogenicity. All renal moieties were hydronephrotic, and left hydroureter was present.	• Karyotype chromosomes: 46,XY	• Cystourethroscopy and dilation at the urethral narrowing (no discrete valve was visualized). Serial imaging demonstrated reduction in size of the bladder diverticulum and grade 1 left and grade 4 right reflux.
				• Bilateral descended testes in a well-formed scrotum and a posterior skin tag containing a rudimentary urethra with no palpable corporal tissue.	• Voiding cystourethrography (VCUG): Revealed bilateral grade 5 vesico ureteral reflux, mild bladder trabeculation, and a proximal urethra caliber change, concerning for posterior urethral valves. Posterior to the bladder was a dilated sac, which communicated below the bladder neck and served as the insertion point of the left ureter. It was felt to represent a periureteral diverticulum or a dilated utricle receiving an ectopic left ureter	• Last creatinine was 0.4 mg/dL (after bilateral ureteral reimplantation and diverticulectomy procedures)	• While bilateral hydronephrosis resolved, right renal atrophy and echogenicity progressed; differential function on that side is 26%.
				• Anteriorly displaced anusm	• Cranial, thoracoabdominal, and spinal imaging were normal.		• Bilateral ureteral reimplantation and diverticulectomy procedures, because recurrent urinary tract infections.
					• No uterus and ovaries were visualized.		
	Case 2: Neonate	-	Penile agenesis	• Bilateral descended testes in a well-formed scrotum and a posterior skin tag containing a rudimentary urethra and no corporal tissue on palpation.	• Ultrasound: Revealed right-sided moderate hydroureteronephrosis and duplication but no left-sided hydronephrosis	• Karyotype chromosomes: 46,XY	• At 23 months, patient underwent cystourethroscopy: Mild bladder trabeculation, a bladder diverticulum, and a posterior urethral valve were detected, and the valve was ablated.
					• VCUG: Revealed a patulous bladder neck, a right-sided bladder diverticulum, a prostatic utricle, and no vesicoureteral reflux.		• Six weeks later, patient underwent an intravesical excisional tapered right ureteral reimplant and paraurethral diverticulectomy.
					• Nuclear imaging: Normal left renal uptake and excretion but delayed right radiotracer uptake and excretion, consistent with obstruction.		• Patient underwent ureterolysis because the progressive postoperative hydronephrosis, cortical thinning and delayed radiotracer uptake and excretion,
					• Cranial, thoracoabdominal, and spinal imaging were normal, and neither imaging nor surgical inspection revealed Mullerian structures.		• Right-to-left transureteroureterostomy at age 4.
					• Interim ultrasound showed worsening of his right-sided hydroureteronephrosis.		
	Case 3: neonate	-	Clitoral absence and urinary tract dysplasia	• Fused, well-formed labia majora. Posterior to this was a skin tag. This skin tag was fleshy on palpation, without any underlying corporal tissue (clitoral absence).	• Ultrasound: Revealed A right dysplastic kidney, a left multicystic dysplastic kidney, and nonvisualization of the bladder.	• Karyotype chromosomes: 46,XY	• This patient had end-stage renal disease (ESRD) from birth and started peritoneal dialysis within the first few weeks of life
				• Underneath the skin tag was a stenotic urogenital sinus 1.5 cm in length. Anus was displaced slightly anteriorly and was somewhat patulous in appearance.	• Ultrasound: Demonstrated a normal uterus and ovaries, which was later confirmed on laparoscopy, along with visual confirmation of fallopian tubes.	• Last serum creatinine was 0.6 mg/dL. (after Creation of an ileocecal neobladder and Mitrofanoff)	• Deceased-donor renal transplant at age 3, with a temporizing ureterostomy.
					• Endoscopy and retrograde imaging of the urogenital sinus: Beyond the area of stenosis, a vagina was seen posteriorly. Anteriorly was a miniscule pouch and right refluxing ureter. No left ureter was identified, consistent with previously identified left multicystic dysplastic kidney. These findings were thought to be consistent with right ureteral ectopia and left ureteral atresia, resulting in either bladder maldevelopment or atresia		• At age 5, creation of an ileocecal neobladder and Mitrofanoff, with concomitant non-refluxing implantation of her transplant ureter.
12. F.A. Talebpur et al., 2016 [13]	Neonate, H-0, gestational age 31 weeks	2 kg	Penile agenesis	• No phallus was seen in baby via clinical examination.	•Urinary endoscopic examination: Urine was mixed with meconium discharged into dentate line proximity.	• Karyotype chromosomes: 46,XY	• Cystostomy was done the day after birth.
				• Scrotum wrinkle and testicular descent was revealed normal.	• Abdominal and pelvis ultrasonography: Severe hydronephrosis and significant diminished left renal cortical thickness along with tough dilation and cystic pelvis were spotted. Mild to average hydronephrosis was seen in the right kidney with mild reduced cortical thickness and pelvis dilatation.		• The patient was discharged for penis reconstruction treatment plan
				• Anus was in normal position.	• Rectum-injected contrast media cystogram: Left vesicoureteral reflux was observed and urethrorectal fistula was verified.		
				• Urinary opening wasn’t seen in perineum.			
				• Urine was mixed with meconium.			
				• Examining hip, there was limitation in hip abduction.			
13. D. Sharma et al., 2015 [14]	Neonate	3 kg	Aphallia with urorectal septum malformation sequence (URSMS)	• Examination revealed an infant with respiratory distress and also with absent penis.	• Only one antenatal scan available: Showed amniotic fluid index of 2, suggestive of severe oligohydramnios.	-	• No active intervention was done, as parents were not willing for any aggressive management and opted for minimal supportive care.
				• The scrotum was well developed with good amount of rugae and bilateral testes were palpable			
				• The infant was noted to have imperforate anus (high anal opening) and single umbilical artery.			
14. Z. Demirer et al., 2015 [15]	22 year old	-	Penile agenesis	• Physical examination: Agenesis of penis, normal scrotum, bilateral normally positioned testis and vas deferens,	• Ultrasonography showed: The absence of left kidney, superior segment of ureter and prostate gland. also seen Left ureterocele, increased bladder wall thickness due to persistent urinary tract infections and dilated right ureter	• Buccal smear was consistent with male genotype.	• The patient and his parents were informed about the masculinizing operation, and they refused any surgical intervention because of their religious beliefs.
				• Urination through rectum	• Cystourethrogram performed through rectosigmoidoscopy: Showed normal appearing bladder, a vesicorectal fistula opening to the anterior wall of the rectum, left ureterocele and grade 2 right vesicoureteral reflux.	• Karyotype chromosomes: 46,XY.	
				• Well male secondary sexual characteristics,	• 99mTc-Dimercaptosuccinyl acid (DMSA) renal scyntigraphy, computerized tomography (CT), urography and magnetic resonance imaging (MRI) were performed and also confirmed USG and Cystourethrography findings.	• All serum hormone levels were normal for an adult male.	
				• Prostate gland was not palpable at digital rectal examination			
15. A. Joshi et al., 2015 [16]	11 month old, GA 39 weeks	-	Aphallia	• Physical examination at birth revealed a well-developed newborn with the absence of the penis and urethra.	• Ultrasonography of the abdomen at birth: Unable to visualize the course of the urethra, it was noticed that when the bladder emptied, there appeared to be more fluid in the rectum, suggesting that it emptied directly to the rectum	• Laboratory investigations were normal, including luteinizing hormone, follicle-stimulating hormone, and testosterone levels.	• The default plan before reconstruction has been to perform a vesicostomy if the patient develop bladder outlet obstruction or a urinary tract infection.
				• The testes were descended bilaterally with normal scrotum and median raphe.	• Magnetic resonance imaging of the abdomen and pelvis at the age of 2 months: Rrevealed increased T2 signal posterior to the base of the bladder, which appeared to extend toward the region of the lower rectum, suggesting ectopic urethral insertion to lower rectum. There was moderate left pyelocaliectasis and hydroureter. In addition, a spinal cord syrinx was identified	• Karyotype chromosomes: 46,XY.	• The patient has not been placed on prophylactic antibiotics, as there has been no evidence of urinary tract infection
				• The anus was patent with no perianal urethral opening identified	• Urinary tract ultrasonography at the age of 9 months: Demonstrated increasing hydroureter without hydronephrosis.		• The patient will undergo urethral exteriorization via perineal urethroplasty around the age of 12 months, followed by pseudophallus construction at the age of 18 months.
					• A suprapubic puncture cystogram: Revealed no evidence of vesicoureteral reflux and no bladder outlet obstruction and better characterized the urethrorectal fistula.		
					• MAG 3 furosemide washout scan: Demonstrated normal drainage of each renal unit, conferring a diagnosis of a nonobstructed left megaureter		
16. S. Aslanabadi et al., 2015 [17]	Neonates, GA 38 week	3450 g	Aphallia with right kidney hypoplasia and leftkidney dysplasia	• The physical examination demonstrated a well-developed male, except for complete absence of a phallus or corporal tissue.	• Ultrasound examination: Revealed a small sized right kidney (18 × 20 mm) without any stones or hydronephrosis. Left kidney was also dysplastic and a cystic mass (17 × 29 mm) was detected within the dysplastic kidney.	• The patient had further creatinine rise and renal failure due to his dysplastic left kidney and hypoplastic right kidney on the following days.	• The parents of the neonate did not give consent for the treatment of the condition.
				• The urine passage was through the rectum by a urethrorectal fistula located in the rectum.	• The ureters and bladder were normal.		• The neonate continued to have creatinine rise and glomerular filtration rate (GFR) decline
				• The scrotum was normal and the neonate had bilaterally descended testes.			• Severe renal failure in fifth day of admission.
				• Anus was placed normally			
				• The urethral opening was not visible anywhere in the perineum.			
17. P. Bahe et al., 2016 [18]	6 days old	2490 g	Complete penile agenesis	• Absence of the penis.	• Ultrasound scan of the abdomen: Suggestive of bilateral hydronephrosis and hydroureter.	• Sepsis and acute kidney injury (serum creatinine 128 mmol/L, serum sodium 165 meq/L, serum potassium 5 meq/L)	• The hydronephrosis was managed with a vesicostomy to relieve ureteric obstruction.
				• Presence of an abnormal common opening for urine and stool.		• Severe metabolic acidosis, severe hyperkalaemia (serum potassium 9 meq/L) and azotaemia (serum creatinine 255 mmol/L).	• Peritoneal dialysis for 48 h and given supportive care.
				• Loose stools and severe dehydration.			• A urinary infection with Klebsiella species was found and treated with appropriate antibiotics
				• Tachypnoeic with a tachycardia, feeble pulse, sunken eyes and reduced urine output.			• Urologist opinion was sought and reconstructive surgery was planned but unfortunately the infant did not return.
18. M. Bothra et al., 2012 [19]	4 years old	-	Complete penile agenesis	• He passed both stools and urine through the anal opening.	• Ultrasonography and intravenous urogram: Showed bilateral grade 3 hydronephrosis.	• Renal function was normal.	• Immediate surgical intervention was planned in view of the hydronephrosis and renal scarring. The urethra was dissected free from the anterior rectal wall till the neck of the bladder, and a perineal urethrostomy was performed.
				• The urine was grossly clear and not mixed with stools.	• Renocystography using 99 m Tc: Showed reflux of radiotracer from bladder to left ureter.	• Serum testosterone, FSH and LH levels were appropriate for age.	• No definitive genital reconstruction surgery was done at this time.
				• The child had a well-developed rugose scrotum with complete absence of the phallus.	• Dimercaptosuccinic acid (DMSA) scan: Left kidney was noted to be smaller with scarring at upper pole.	• Asymptomatic bacteriuria ( E.coli > 10 5 CFU/mL) was detected, possibly due to fecal contamination of urine.	
				• Both testes were present in the scrotal sac and were normal in volume (2 mL bilaterally) and texture.	• MRI of the abdomen and scrotum: Revealed absent penile tissue, normal size and signal intensity of testes, dilated right ureter and fullness of right pelvicalyceal system with no other congenital anomaly.		
				• The anal opening was placed normally. No separate urethral opening was visible on the perineum or anal verge.	• Retrograde radiographic study with contrast instillation into anal opening opacified the rectum and sigmoid colon, as well as the urinary bladder, indicating a fistulous connection.		
19. Willihnganz et al., 2012 [20]	3 month old	-	Aphallia	• High-grade fever along with excessive crying and straining at micturition.	• Ultrasonography of the abdomen: Showed a small left kidney with moderate hydro-ureteronephrosis and an irregularly thickened urinary bladder.	• Karyotype chromosomes: 46,XY	• Patient discharged on antibiotic prophylaxis and anticholinergic
				• Testes were bilaterally descended in a well-developed scrotum	• Renal scintigraphy: Impaired cortical function, renal scars, and slow clearance on the left side with near-normal function on the right side.	• The renal function tests were normal	• He was operated by anterior sagittal transscrotal approach in a lithotomy position.
				• The stenosed perineal urethral orifice opened in the anterior wall of the anorectum.			• The urethral opening was separated from the anorectum and transposed at the penoscrotal junction as perineal urethrostomy.
							• De Castro Phalloplasty with 4 × 5-cm lower abdominal quadrangular flap was performed with suprapubic cystostomy.
20. P Arunachalam et al., 2012 [21]	Neonate, H-3	2200 g	Aphallia and cloaca exstrophy	• Patient’s had omphalocele, 2 hemibladders with elephant trunk appearance of the bowel segment, imperforate anus, bifid scrotum with descended testes, and absent phallus.	• Biopsy was taken from the tip of phallus, Histopathologic examination: Revealed glans penis with corpus spongiosum made up of irregular vascular channels separated by fibrous stroma and corpus cavernosum with thick interanastomosing vascular structures. Urethral meatus lined by stratified squamous epithelium was present in the center of corpus spongiosum.	• Karyotype chromosomes: 46,XY	• The infant was stabilized and then taken up for surgery. There was a 12-cm length of distal colon, and the bowel patch was tubularized and the end brought out as colostomy
				• The base of the bladder was intact with a phallus-like structure without skin covering entrapped in the base of the bladder.			• The infant continued to deteriorate in the postoperative period because of multiple complications and died.
21. A.D. Kane et al., 2011 [22]	Neonate, 8 hours after birth	-	Absence of penis	• Clinical examination showed a good general state, an absence of penis and urinary meatus.	• Direct cystourethrogram: Showed a lower-end urethro- rectal fistula and a grade III right vesicorenal reflux.	• Full blood count, blood ionogram, hepatic and renal state were normal.	• Cystostomy was performed 4 days after birth followed by anterior transposition of the urethra at the 10th month
				• Palpation revealed the presence of the gonad inside the scrotum.	• The other imaging investigations (standard abdomen X-ray, abdomen, and heart echography and the CT scan) were normal	• Cytobacterial examination of the urine was normal.	• He presented an evisceration at day 15 post-operatively, which was successfully repaired.
				• The anus was in normal position, permeable, with the presence of meconium mixed with urine.		• Karyotype chromosomes: 46,XY	• At the age of 1 and a half years, the cosmetic aspect of the perineum was satisfactory with a well-anchored urethra at the level of the median raphe and the anus was at its normal position.
							• It was planned that the child would undergo phalloplasty at the age of puberty.
22. K.H. Willihmganz et al., 2012 [20]	Premature neonate, GA 36 weeks	-	Aphallia	• His prenatal course had been complicated by oligohydramnios.	• Additional imaging studies found: A multicystic horseshoe kidney with abnormal architecture and 2 distorted collecting systems suggesting a multi cystic dysplastic kidney. No ureters or bladder were identified.	-	• Peritoneal dialysis was begun shortly after birth due to renal failure.
				•Initial examination the infant was found to have aphallia with a normal scrotum, bilateral palpable testes and a very small perineal fistula for a urethra.			• At 22 months of age, the boy underwent a cadaveric renal transplant with cutaneous ureterostomy for diversion.
							• After multiple episodes of stenosis of the ureterocutaneous anastomosis, at 30 months old, he underwent takedown of the ureterostomy and creation of a catheterizable stoma from terminal ileum and a modified Florida pouch using the right and transverse colon.
							• At about 5 years of age the family agreed to proceed with construction of a neophallus using the De Castro abdominal flap technique.
23. H. Wang et al., 2011 [23]	31 years old man	-	Absence of phallus	• Physical examination looked normally developed and examination of the heart, lungs, abdomen, head and neck were all normal.	• Ultrasonography: Showed a thick-walled bladder was present.	• Karyotype chromosomes: 46,XY.No AZF or SRY gene deletion was detected by polymerase chain reaction analysis.	• Bilateral epididymectomy and right nephrectomy were done at the age of 28 years because of congenital dysplasia.
				• External genitalia examination found two descended and palpable normal testicles with the complete absence of a phallus or corporal tissue.	• An intravenous pyelogram: Left solitary kidney without hydronephrosis or ureteric dilation.	• Serum reproductive hormone profiles were carried out (testosterone, prolactin, estradiol luteinizing hormone/follicle stimulating hormone assays) and were within normal levels	• A total phallic reconstruction was not recommended, because he had the right kidney removed, and another operation was a major challenge.
				• Digital rectal examination revealed the urethral meatus could be detected on the anterior wall of the rectum posterior to the sphincter with the depth of 1 cm.	• Voiding cystography: Oval-shaped urethra was observed between the coccygeal apex and the bladder in the lateral position.	• Testicular sperm extraction showed large number of progressive sperm in the fluid smear	• Therefore, we recommended that he should find a woman without sexual desire as his partner.
					• MRI: Provided distinct visualization of penile agenesis and the urethra opening to the anterior wall of the rectum posterior to the sphincter, accurately identifying the hypoplastic prostate and determining the course of the urethra and the surrounding musculature		
24. M.G. Blanluet et al., 2011 [24]	Neonate, terminate of pregnancy at 35 weeks	-	Aphallia, with imperforate anus, bilateral renal dysplasia and complete right lung agenesis	• Fetopathological examination showed: Anormotrophic boy, with imperforate anus, aphallia with normal scrotum, and bilateral clubfeet.	• Severe oligohydramnios was discovered at 32 weeks, An ultrasound scan disclosed severe bilateral renal dysplasia with no residual renal function.	• Karyotype chromosomes: 46,XY	• Termination of pregnancy was performed at 35 weeks.
				• Potter sequence was present, with dysmorphic features; large, rotated ears, a short compressed nose, and deep subocular grooves.			
				• A single umbilical artery was present with fusion of a right and a thin left umbilical cord arteries.			
				• The rectum was blind and dilated.			
				• Kidneys were cystic, the left kidney being larger than the right. Histologic analysis showed fibrous dysplastic cystic kidneys with few tubules or glomeruli present.			
				• The bladder was hypoplastic with urethral atresia due to the aphallia. Both vasa deferentia were present, and opened into the ipsilateral ureter, as a persistence of urogenital horns.			
				• The left lung was normal with two lobes, the right lung was absent, with a thin pleura adherent to the right part of the thorax.			
				• The right main bronchus was present, but there was no right pulmonary artery or pulmonary vein.			
				• The heart showed myocardial hypertrophy, frequently associated with severe oligohydramnios			
				• Testes were normal, scrotal for the left testis and inguinal on the right.			
25. K.N. Rattan et al., 2009 [25]	Case 1: Neonate, H-1 aterm	2000 g	Absence of phallus	• Absence of the phallus and no anal opening. The urethral opening was located on a skin appendage near the anal pit.	• Ultrasound study of the abdomen: Left solitary kidney and normal urinary bladder.	• Buccal smear was negative for Barr bodies	• The baby underwent a sigmoid colostomy. The infant was discharged with definitive reconstruction planned at a later date.
				• Well-developed scrotum was present in the normal position, but the gonads were not palpable	• Plain invertogram radiograph: Suggested a high type of anorectal malformation		• Posterior sagittal anorectoplasty and urethral dilation were done at 10 months of age. • The child is now 13 months old. He has normal renal parameters and a normally functioning left solitary kidney.
							• We intend to do phallic reconstruction at a later time.
	Case2: Neonate	-	Absence of phallus	• Patient appeared severely ill and dehydrated. Sepsis was suspected.	• Ultrasound of the abdomen: Right ectopic kidney in the pelvis. The left kidney and bladder were normal.	• Buccal smear was negative for Barr bodies	• The baby died because of septicemia before any definitive therapy. Consent for autopsy was refused by the parents.
				• Perineal examination revealed a normal anus with a fleshy tag of skin and small pinpoint opening in front and outside the anal verge that discharged urine in a very thin stream.		• Chromosomal studies were not done	
				• Phallus absent from its normal site, scrotum was normally positioned and well developed. No gonads were palpated on either side.			
	Case 3: 6 month old	4000 g	Absence of phallus	• Phallus was absent. The scrotum was well developed, and bilateral testes were palpable.	• Antegrade cystography: Fistulous opening between posterior urethra and rectum	• Buccal smear was negative for Barr bodies	• The parents refused further treatment. The child has been lost to follow-up.
				• The anal opening was located normally with urine coming from it.			
26. Y. Nakano et al., 2009 [26]	Neonate, GA 32 weeks	1440 g	Penile agenesis associated with urorectal septum malformation sequence (URSMS),	• The infant had a small and low-set exomphalos without any lower abdominal wall defect;	• Cardiac and cranial echograms: Normal results.	• Routine laboratory examinations showed normal results	• The infant underwent surgery, including a cystostomy (vesicocutaneous fistula) and colostomy.
			Covered cloacal exstrophy with SSBS	•The bladder was covered with normal skin and subcutaneous tissue.	• Simple radiograph of the abdomen: Revealed wide pubic diastasis.		• Intra operative finding: The small bowel was only 14-cm long (severe short bowel syndrome [SSBS]), a patent urachus without exomphalos, The colon, cecum, and appendix were duplicated.
			Patent urachus	• The genital anomalies detected were penile agenesis and bifid scrotums; the testes on both sides were palpable.	• Abdominal ultrasound after birth: Revealed normal-sized kidneys without hydronephrosis.		• The SSBS had gradually caused liver dysfunction with cholestasis, the infant died from hepatic insufficiency on day 132 after birth
				• Urine and stool were excreted around the umbilical region through the urachus.	• Cystogram through the urachus: Revealed that the bladder was open to the intestine		
				• The infant had no spinal defect, but had mild bilateral radial dysplasia.			
27. Al. Shamsa et al., 2008 [27]	18 month old child	2500 g	Aphallia associated with urethrorectal fistula and stones in the bladder and urethra	• The child looked normally developed and examination of the heart, lungs, abdomen, head and neck were all normal.	• Renal ultrasonography, plain x-ray of the abdomen, intravenous pyelogram, chest x-ray, wrist x-ray and antegrade cystography were normal except for the presence of three large bladder and three small urethral stones on plain x-ray.	• Urine analysis and culture confirmed asymptomatic bacteriuria.	• On the seventeenth day of age he had undergone open cystostomy at the district hospital.
				• Phallus was absent, the scrotum was normal with two normally descended testes with palpable vas deferens.	• Intravenous pyelogram: Confirmed reflux of contrast media from urethra to the descending colon and rectum.	• Blood biochemistry and routine hematologic tests were normal	• All bladder and urethral stones were disintegrated and aspirated through the cystostomy tract, by Swiss lithoclast and Elixs evacuator
				• No voiding per urethra. The voided urine was mixed with fecal material	•Retrograde urethrocystoscopy: Confirmed the presence of a urethro-rectal fistula	• Karyotype chromosomes: 46,XY	• Following the stone clearance, the patient underwent closure of the fistula using the perineal approach, the urethra was exposed and divided from the urethro-rectal junction.
				• The urethra had an opening into the rectum about 2.5–3 cm distal to the bladder neck.			• The urethra was then dissected free from its underlying connective tissue up nearly to the bladder neck to establish a perineal urethrostomy
							• Patient planned total phallic reconstruction and urethroplasty after attaining puberty.
28. B.C. Reiner et al., 2007 [28]	Neonate, H-2, GA 39 weeks	-	Ambiguous genitalia	• The physical examination demonstrated a well-developed male with a rudimentary genital bud (5 mm length).	• The voiding cystography: Demonstrated bilateral grade II vesicoureteral reflux, normal bladder and posterior male urethra.	• Karyotype chromosomes: 46,XY	• Parent decided the child should be raised as a female.
				• The scrotum was normal with a median raphe and contained two testes.	• Ultrasound and MRI examination: Showed a complete absence of Mullerian structures and two small erectile structures in the genital bud.	• Hormonal investigations were performed (testosterone, inhibine B, antimüllerian hormone, dihydrotestosterone, luteinizing hormone (LH)/follicle stimulating hormone (FSH) assays) and were normal.	• The patient underwent bilateral orchiectomy at 4 months of age, followed by a clitoral reconstruction, a flap vaginoplasty and a labioplasty.
				• The urethral meatus was located at the base of the genital tubercle.		• Amplification by polymerase chain reaction of the 5a-reductase and the androgens-binding receptor were normal.	• The patient will require the creation of a functional vagina at puberty.
				• There was a normally located anus.			
29. P. Jal Chibber et al. 2005 [29]	16 years old	-	Aphallia	• Examination revealed a normal child with an absent penis, had a normal scrotum with bilaterally descended testis.	• Ultra-sonography and IVP: Bilaterally normal kidneys.	-	• Medical advice was not sought by the parents till his age of 16 years, and after discussed with parents, proper consent for phallic reconstruction was taken.
				• Urine was passing through the anal opening.			• Implantation of penile prosthesis is planned at a later date.
				• On examination the urethra was found to open just above the anal verge.			
30. C.B. Threatt et al., 2003 [30]	Neonates, H-2, GA 40 weeks	-	Ambiguous genitalia	• Normally developed male, except for the complete absence of a phallus or corporal tissue.	• Renal/bladder sonography: Right solitary kidney without hydronephrosis or ureterectasias. The bladder was markedly thickened, with fluid visible in the rectum, suggestive of a rectourinary fistula.	Karyotype chromosomes: 46,XY	• At 10 months of age, the child was taken to surgery to separate the urinary tract from the rectum.
				• No urethral meatus was visible. Both testes were descended and palpable in the normally developed scrotum.	• Voiding cystourethrography using suprapubic instillation of 35 mL of contrast: Demonstrated a competent bladder neck. The patient voided through a shortened urethra coursing posteriorly into the rectum approximately 2 cm from the anal verge		• Phallic construction is planned for late childhood before puberty.
				• Stool production was noted to be watery, consistent with a mixture of urine and feces	• Barium enema: Showed normal distension of the lower rectum without demonstration of reflux into the urethra.		
31. A.O. Cifci- 1995 [31]	6 years old	-	Penile agenesis and bilateral undescended testis	• The physical examination showed a well-developed boy with no penis and with bilateral undescended testis that were easily palpated in the inguinal region.	• Intravenous pyelogram: Showed an ectopic hydronephrotic kidney on the right side, without any visible left kidney or ureter.	• Karyotype chromosomes: 46,XY	• The patient underwent bilateral orchiopexy, followed by perineal transposition of urethra to avoid chronic urinary tract infection.
				• The scrotum was normal with a median raphe.	• Voiding cystourethrogram via the rectal urethral opening: Showed a normal bladder	• Chromatin negative buccal smear.	• Postoperatively, appropriate antibiotics were administered and the patient discharged without any complaints.
				• There was a normally placed anus with an anterior skin tag.	• Fluoroscopic examination: Showed that the patient voided approximately 8O% of the bladder contents voluntarily, without difficulty.	• Results of laboratory investigations (including a complete blood cell count and liver and kidney function tests) were normal.	• Patient did not attend his outpatient appointments and was readmitted after 6 years, at age 12, with signs and symptoms of chronic renal failure. Drug therapy was begun, patient died at age 13 of sepsis and uremia.
				• The urine exited from the anus, through the urethral meatus on the anterior rectal wall.		• Urinalysis and urine culture: Showed persistent infections of Escherichia coli and Pseudomonas	
				• Globular mass suggestive of an ectopic hydronephrotic kidney was palpated on the right side of the abdomen.			
32. M.C. Carr- 1994 [32]	Neonate, H-0, GA 35 weeks	2400 g	Aphallia and cloaca exstrophy	• Abnormalities on physical examination included an omphalocele, a meconium stained umbilical cord that was markedly ectatic, a bifid scrotum with palpable gonads and absent phallus separation of the pubic rami, and urethral or rectal openings on the perineum.	• Ultrasonographic evaluation at 26 weeks’ gestation: Examination of the fetal genitalia revealed a normal scrotum and testes with no phallus present, the kidneys appeared normal, no bladder was visualized, an omphalocele was present with a low insertion of the umbilical cord Amniotic fluid volume was normal	-	• Decision was made to reassign the neonate to a female gender
					• Pathologic examination of the bladder mass: Consistent with a hamartoma consisting of squamous mucosa surrounding erectile tissue.		• The infant was taken to the operating room 6 hours after birth and underwent exploratory laparotomy; A vesicostomy was created and gonadectomies performed.
33. S.J. Skoog et al., 1989 [33]	Case 1: Neonates, H-2	-	Penile agenesis	• Absence of the phallus with a skin tag on the anterior anal verge.	• The excretory urogram (IVP): Revealed normal upper tracts.	• Buccal smear was negative for chromatin.	• Bilateral orchiectomy, urethral transposition and labial construction were done when the child was 2 weeks old.
				• The urethral meatus was not visible.	• The voiding cystourethrogram: Demonstrated grade II left vesicoureteral reflux, a normal bladder and drainage of contrast medium from the distal urethra into the rectum (pre-sphincteric meatus).	• Karyotype chromosomes: 46,XY	• Oral antibiotics and periodic urethral dilation.
				• The scrotum was entirely normal and contained 2 gonads.			
				• The remainder of the physical examination was normal.			
	Case 2: 4 weeks old, GA 40 weeks	-	Penile agenesis	• Fully formed scrotum contained 2 normal gonads and a right hydrocele.	• Renal scan was normal	• Buccal smear was negative for chromatin.	• Bilateral orchiectomy, urethral transposition and labial construction with scrotal skin were performed.
				• A skin tag and small proboscis were noted at the anterior anal verge.	• Cystogram: Demonstrated a normal bladder without evidence of fistulous communication with the rectum.	• Karyotype chromosomes: 46,XY	
				• A small opening at the base of the skin tag was recognized as the urethral meatus.			
	Case 3: 1 week old,	-	Ambiguous genitalia and renal insufficiency	• Absent phallus and a peculiar tuft of tissue at the anterior anal verge.	• A renal and abdominal sonogram: Demonstrated a large retrovesical cystic mass with anterior bladder displacement. The right kidney and ureter were dilated but a left kidney could not be demonstrated.	Admission serum creatinine was 1.2 mg./dl. (normal 0.9 to 1.5) and mild hyperchloremic acidosis	• A 5F feeding tube was inserted into the bladder and the serum creatinine decreased to 0.5 mg./dl.
				• A small amount of erectile tissue was present anteriorly, which was engulfed by a normal scrotum.	• Diethylenetriaminepentaacetic acid renal scan with delayed images: Defined the hydronephrotic right kidney but no function was noted on the left side. Delayed images revealed nuclide in the retrovesical cystic mass		• At 10 days after birth a dysplastic left kidney that drained into a midline cystic mass was resected. The mass communicated distally in the area of the prostatic urethra.
					• Voiding cystourethrogram: visualized a trabeculated bladder with faint contrast medium in the dilated retrovesical mass and a type III posterior urethral valve.		• Vesicostomy was performed to provide unobstructed drainage of the right kidney
							• Bilateral scrotal orchiectomy then was performed.
							• The scrotal halves were brought into the midline to simulate labia majora and the glans-like tubercle was left intact at the superior margin of the labial cleft
							• Patient underwent antegrade fulguration of the posterior urethral valve with closure of the vesicostomy.

### Demographics

The most cases were reported in the USA which were 31% (n:13) of all cases [2, 7, 8, 12, 16, 30, 32–34], followed by India with 21% (n:9) [14, 18, 19, 21, 25, 29, 34] Iran with 12% (n:5) [5, 13, 17], China with 7.3% [1, 6, 23], Turkey with 4% (n:2) [15, 31], France with 4% (n:2) [23, 27], Japan with 4% (n:2) [26, 27], Morocco with 4% (n:2) [9, 10], Mexico with 2% (n:1) [3], UK with 2% (n:1) [11], and Senegal with 2% (n:1) [22].

The majority of cases involve males with 97% (n:40) of all cases (1–3,7–29), and 2% (n:1) was female [2]. Of the 35 cases, the majority with 48% (n:20) cases were term neonates [2, 3, 8–12, 14, 17, 18, 21, 22, 25, 28, 30, 33], while 19% (n:8) were preterm neonates [2, 13, 24, 26, 32, 34], 9% (n:4) were infants [1, 16, 25, 34], 9% (n:4) were under five [5, 7, 19, 27],and 12% (n:5) were over 5 years old [6, 15, 23, 29, 31]. The patient with the lowest gestational age was 31 weeks [13], with the oldest patient ‘s age was 31 years old [23].

Only 14 cases reported weight. As many as 19% (n:8) of cases reported low birth weight neonate [13, 18, 21, 25, 26, 32], and 14% (n:6) of cases were normal birth weight neonates [9, 11, 14, 17, 25, 27]. The case with the lowest birth weight was 1,440 g [26] and the case with the largest birth weight was 4,000 g [25]. All characteristic data are shown in Table 2.

**Table 2:** Characteristics of all patients

Demographic	Variable	Result
Country	USA	31% (n:13)
	India	21% (n:9)
	Iran	12% (n:4)
	China	7.3% (n:3)
	Turkey	4% (n:2)
	France	4% (n:2)
	Japan	4% (n:2)
	Morocco	4% (n:2)
	Mexico	2% (n:1)
	UK	2% (n:1)
	Senegal	2% (n:1)
Sex	Male	97% (n:33)
	Female	2% (n:1)
Age	Preterm Neonate	22% (n:8)
	Term Neonate	48%(n:17)
	Infants	11% (n:4)
	1–5 years old	5% (n:2)
	≥ 5 years old	11% (n:4)
Birth Weight in neonates	Low birth weight	19% (n:8)
	Normal birth weight	14% (n:5)

### Presentation

On physical examination, as many as 95% (n:39) cases reported scrotum development, and 2% (n:1) case reported well-formed labia major [2]. From 39 cases, 85% (n:35) cases reported normal scrotum development [1–3, 5–19, 22–25, 27–31, 33, 34], and 9% (n:4) reported bifid scrotum [13, 21, 26, 32]. Almost all male cases reported testicular development (with 39 of the 40), with 82% (n:34) with descending testis [1–3, 5–7, 9–23, 25–30, 32–34] and 12% (n:5) with undescendent testes [8, 24, 25, 31].

The majority of the patients’ anus was in a normal position in 78% (n:32) cases [1–3, 5–7, 9–13, 15–20, 22, 27–33], with 2% (n:1) an anteriorly displaced anus [2], 17% (n:7) with imperforated anus [8, 14, 21, 22, 24–26], and 2% (n:1) with funnel anus [11]. As many as 22% (n:8) of the cases were found to have skin tags in the perineal area (2,21,27,29).

Other findings were 4% (n:2) cases with sepsis [18, 24], 7% (n:3) cases with respiratory distress [2, 13, 14], 2% (n:1) case with Potter sequence [24] and 4% (n:2) cases with clubfoot [13, 24]. All of these presentations are presented in Table 3.

**Table 3:** Physical examination finding in all cases

Physical Exam	Finding	Reported	Result
Scrotum		95% (n:39)	
	Normal		85% (n:35)
	Bifid Scrotum		9% (n:4)
Labium		2% (n:1)	
	Normal		2% (n:1)
Testis		95% (n:39)	
	Descending testes		82% (n:34)
	Undescendent testes		12% (n:5)
Anus		100% (n:35)	
	Normal position		78% (n:32)
	Anteriorly displaced anus		2% (n:1)
	Imperforated		17% (n:7)
	Funnel Anus		2% (n:1)
Other	Sepsis		4% (n:2)
	Respiratory distress		7% (n:3)
	Potter sequence		2% (n:1)
	clubfoot		4% (n:2)

### Laboratory examination

Laboratory tests were conducted differently in each case. All such tests are presented in Table 4. Karyotype examination was performed in 29 cases. Almost all of these cases reported karyotype XY with 68% (n:28) [1, 2, 6, 8–13, 15, 16, 18, 20–25, 27, 28, 30, 31, 33], and 2% (n:1) case reported 46XX karyotype [2].

**Table 4:** Laboratory examination in all cases

Laboratory Test	Laboratory Data	Reported Cases	Result
Karyotype		70% (n:29)	
	XY		68% (n:28)
	XX		2% (n:1)
Kidney Function		39% (n:16)	
	Normal		21% (n:9)
	Abnormal		17% (n:7)
Urinalysis		12% (n:5)	
	Symptomatic Bacteriuria		4% (n:2)
	Asymptomatic Bacteriuria		7% (n:3)
Hormonal profile		19% (n:8)	
	Normal		19% (n:8)
Others Lab Exam	Metabolic acidosis		7% (n:3)
	Sepsis		2% (n:1)
	AKI		2% (n:1)
	Kidney Failure		7% (n:3)

Only 16 cases reported kidney function [2, 6, 9–11, 13, 14, 16–20, 27, 33], indicating 21.9% (n:9) cases had normal kidney function results [2, 6, 9, 11, 16, 19, 27, 33, 34], and 17% (n:7) cases had abnormal kidney function results [2, 10, 11, 13, 17, 18, 27]. In 5 cases with abnormal kidney function, 5% (n:2) cases were found with renal dysplasia [2, 17], 2% (n:1) with atrophic renal due to multicystic bladder [13], 2% (n:1) with a single left urethra with rectobladder fistula draining to an anal funnel [11], and 2% (n:1) with sepsis due to urinary tract infection [18].

As few as 12% (n:5) cases reported bacteriuria with 4% (n:2) with symptomatic bacteriuria [18, 34], and 7% (n:3) with asymptomatic bacteriuria [19, 27, 31]. Only 8 cases reported hormonal profile and all cases showed normal results [3, 10, 15, 16, 18, 19, 23, 28].

Other lab examinations showed 7% (n:3) of all cases had metabolic acidosis [11, 18, 33], 7% (n:3) had kidney failure [13, 17, 20], while 2% (n:1) had sepsis and acute kidney injury due urinary tract infection [18].

### Imaging investigation

Most cases used postnatal ultrasound in 75% (n: 31) cases [1–3, 5–19, 23, 25–30, 33, 34] followed by 46% (n:19) using cystography [2, 10, 13, 15, 16, 22, 23, 25–28, 30, 31, 33], 22% (n:9) using urography [2, 7, 15, 19, 23, 27, 29, 31, 33], 22% (n:9) using magnetic resonance imaging (MRI) [7, 8, 11, 12, 15, 16, 19, 23, 28], 17% (n:7) using nuclear imaging [2, 15, 16, 19, 33, 34], 17% (n:7) of cases using prenatal ultrasound [8, 12, 16, 24, 26, 28, 32], 14% (n:6) used computerized tomography (CT) scan [1, 1, 3, 5, 6, 15, 22], and 12% (N5) used endoscopy [2, 12, 27, 33].

Some studies used other imaging studies to exclude other system anomalies. As many as 17% of cases (n: 7) used echocardiogram [1, 3, 7, 13, 14, 18, 22], 4% (n: 2) used GI contrast [11, 30], and 7% (n: 3) used chest X-ray [14, 24, 27]. Only 1 case reported not using an imaging investigation because the parent did not provide consent [21].

Anomalies were found in the genitourinary system, cardiovascular system, respiratory system, digestive system, and muscular system (Table 5).

**Table 5:** Anomalies’ finding in all cases

Affected System	Organ	Anomaly	Reported	Result
Genitourinary	Renal		63% (n:26)	
		Renal Agenesis		12% (n:5)
		Hypoplastic renal		4% (n:2)
		Dysplastic renal		19% (n:8)
		Hydronephrosis		34% (n:14)
	Ureteral		29% (n:12)	
		Reflux Ureteral		26% (n:11)
		Absence		7% (n:3)
		Ectopic Ureteral		2% (n:1)
		Ureterocele		2% (n:1)
	Bladder		21% (n:9)	
		Absence of bladder		7% (n:3)
		Hypoplastic bladder		4% (n:2)
		Posterior bladder diverticulum		4% (n:2)
		Hemibladder		2% (n:1)
		Three bladders		2% (n:1)
	Prostate		4% (n:2)	
		Prostate absence		2% (n:1)
		Hypoplastic prostate		2% (n:1)
	Urethra		21% (n:9)	
		Absence of urethra		9% (n:4)
		Posterior ureteral valve		7% (n:3)
		Urethral stone		2% (n:1)
		Urethral stenosis		4% (n:2)
	Fistula		80% (n: 28)	
		Urethrorectal		46% (n:19)
		Urethroperineal		17% (n:7)
		Vesicorectal		59% (n:4)
		Urachal fistula		4% (n: 2)
		Ureterorectal		2% (n: 1)
Respiratory system	Lung		9% (n:4)	
		right lung absent		2% (n:1)
		pulmonary hypoplasia		2% (n:1)
		pulmonary hypertension		2% (n:1)
		pulmonary hyperplasia		2% (n:1)
Cardiovascular system	Cardiac		12% (n:5)	
		PDA		4% (n:2)
		ASD		4% (n:2)
		tricuspid valve insufficiency		7% (n:3)
		hypertrophic myocardia		2% (n:1)
Digestive system			8% (n:3)	
		Omphalocele		4% (n:2)
		duplicated colon,		2% (n:1)
		Duplicated caecum		2% (n:1)
		Duplicated appendix		2% (n:1)
		short bowel syndrome		2% (n:1)
Musculoskeletal System	Lower Extremity		4% (n:2)	
		clubfoot bilateral		4% (n:2)

The genitourinary system is the most affected system with anomalies in aphallia cases. Fistulas occurred in 80% (n: 29) of cases and were the most commonly reported anomalies [1–3, 5–7, 9–13, 15–17, 19, 22, 23, 25–27, 29–34]. Urethrorectal fistulas are the most common fistula occurred in 46% (n:19) cases [1, 13, 16, 17, 19, 22, 23, 25, 27, 29–31, 33, 34], followed by urethroperineal fistula with 17% (n:7) cases [2, 7, 20, 25], vesicorectal fistula with 9% (n:4) cases [3, 5, 11, 15], urachal fistula by 4% (n: 2) [26, 32], and ureterorectal fistula with 2% (n: 1) case [12].

Other genitourinary anomalies found are in the kidneys, ureters, bladder, prostate and urethra. Renal abnormalities occurred in 63% (n:26) cases. Of the 22 cases with renal anomalies, renal agenesis occurred in 12% (n:5) [11, 14, 15, 25, 30], hypoplastic kidney in 4% (n:2) cases [3, 5], renal dysplasia in 19% (n:8) [2, 12, 13, 17, 20, 23, 24, 33], and hydronephrosis in 34% (n:14) [2, 8–11, 13, 18, 19, 25, 30, 31, 33, 34].

The ureteral anomaly occurred in 29% (n:12) of all cases. From 12 cases reported, ureteral reflux occurred in 26% (n:11) [2, 9, 10, 13, 15, 19, 22, 28, 33], absence of ureter occurred in 7% (n:3) [2, 15, 20], ectopic ureter in 2% (n:1) [2], and ureterocele occurred in 2% (n:1) [15].

Bladder anomalies occurred in 21% (n:9) of all cases [2, 12, 20, 21, 24, 27, 32]. Of the 9 cases, 7% (n:3) with absence of bladder [2, 12, 20], 4% (n:2) with hypoplastic bladder [24, 32], 4% (n:2) with posterior bladder diverticulum [2], hemibladder in 2% (n:1) case [21], and three bladders in 2% (n:1) [27].

Prostate absence occurred in 2% (n:1) of all cases [15], and hypoplastic prostate in 2% (n:1) of all cases [23]. The urethra anomalies occurred in 21% (n:9) [2, 3, 24, 26, 27, 32, 33], involving 9% (n:4) with absence of the urethra [3, 24, 26, 32], 7% (n:3) with posterior ureteral valve [2, 33], 4% (n:2) with urethral stenosis [2, 33], and 2% (n:1) with urethral stone [27].

The anomaly in the respiratory system occurred in 9% (n:4) of all cases [1, 11, 14, 24]. right lung absent occurred in 2% (n:1) case [24], 2% (n:1) case with pulmonary hypoplasia [14], 2% (n:1) case with pulmonary hypertension [1], and 2% case (n:1) with pulmonary hyperplasia [14]. Anomalies in the cardiovascular system were found in 12% (n:5) of all cases [1, 13, 14, 24]. Of the 5 cases, patent ductus arteriosus occurred in 4% (n:2) [1, 13], atrial septal defect in 5% (n:2) [1, 14], 7% (n:3) in tricuspid valve insufficiency [1, 13], and 2% (n:1) with hypertrophic myocardia [24].

In the digestive system, anomalies occurred in 9% (n:3) of all cases. Omphalocele occurred in 4% (n:2) [21, 32], and 2% (n:1) with duplicated colon, caecum, appendix, and short bowel syndrome [26]. In extremity anomalies, clubfoot bilateral occurred in 4% (n:2) of all cases [13, 24].

## Management

Anomalies can be different in each case, causing management to differ in each case (Table 6). As many as 39% (*n* = 16) patients underwent vesicostomy [7–11, 13, 16, 18, 20–22, 26, 27, 32, 33]. Renal transplant and nephrectomy respectively were performed in 2% (n:1) of all cases [2, 23]. Ureteral reimplant was performed in 4% (n:2) [2], urethrostomy in 9% (n:4) [2, 19, 20, 27], and perineal urethroplasty in 9% (n:4) case [6, 16, 19, 27]. As few as 9% (*n* = 4) cases required dialysis [2, 12, 18, 20].

**Table 6:** Management summary in all cases

Organ	Management		Result
Renal	Nephrectomy		2% (n:1)
	Renal Transplant		2% (n:1)
	Dialysis		9% (*n* = 4)
Urethra	Urethrostomy		9% (n:4)
	Ureteral Reimplant		4% (n:2)
	Perineal Urethroplasty		2% (n:1)
Vesica	Vesicostomy		39% (n: 16)
Further management			
(sex determination)	Male		
		Phalloplasty	7% (n:3)
		Penile reconstruction	24% (n:10)
	Female		
		Feminization	12% (n: 5)
Other	Refuse treatment/ lost follow-up		17% (n:7)
	Deceased		12% (n: 5)

Further management in 7% patient (*n* = 3) involved phalloplasty [12, 20, 34], followed by 24% (n:10) that planned for penile reconstruction [6, 10, 11, 13, 16, 22, 25, 27, 29, 30], and 12% (*n* = 5) that chose feminization [28, 32, 33]. The number of patients who refused medical treatment or were lost to follow-up were as many as 17% (n:7) of all cases [1, 13–15, 17, 18, 25], and 12% (*n* = 5) of all patients deceased [13, 21, 25, 31].

## Discussion

Penile agenesis is a very rare genitourinary occurrence with a prevalence of 1 in 30 million births [1]. Aphallia is defined as the absence of a phallus and ectopic urethral opening and associated with well-developed scrotum and bilateral palpable testes [35].

In males, the absence of the penis is characterized by the absence of three penile components: two corpora cavernosa and the corpus spongiosum. Aphallia can also occur in female patients, although it is considered less common and more difficult to diagnose. This is most likely due to a high association with bladder agenesis and intrauterine fetal demise [35].

In this review, the country with the most reported cases of aphallia was the United State [2, 7, 8, 12, 16, 30, 32–34], but regionally the most cases were in Asia. Patients with aphallia generally present at birth [2, 3, 8–12, 14, 17, 18, 21, 22, 25, 28, 30, 33], but in some cases patients come after being older than 1 month, for unknown reasons. All patients were male (1–3,7–29), except for one case where the clitoris was absent in a woman [2]. Most of the previous reports of aphallia were limited to boys, with some reports of clitoral agenesis in girls. This may be related to the underdiagnoses of aphallia in girls, where the absent clitoris is far less conspicuous than an absent penis.

Physical examination in the majority of studies showed that most patients had normal scrotal growth [1–3, 5–19, 22–25, 27–31, 33, 34], normally descended testes [1–3, 5–7, 9–23, 25–30, 32–34], and a normally sited anus [1–3, 5–7, 9–13, 15–20, 22, 27–33]. There can be abnormalities such as imperforated anus [8, 14, 21, 22, 24–26], bifid scrotum [13, 21, 26, 32], and undescended testes which occurred in a minority of cases for unknown reasons [8, 24, 25, 31].

This systematic review shows the various Imaging and laboratory modalities used with aphallia, and almost every case used a different modality. This may be because of the many anomalies that occurred, so it requires a different approach for each case and it should be noted that most cases have multiple anomalies.

Laboratory examination in karyotype examination revealed the majority of the cases are male [1, 2, 6, 8–13, 15, 16, 18, 20–25, 27, 28, 28, 30, 31, 33], and the majority had normal laboratory results. All hormonal profiles were in the normal range [3, 10, 15, 18, 19, 23, 28]. Besides, there are some abnormal laboratory tests on a small portion of cases that involve bacteriuria [18, 19, 27, 31, 34], metabolic acidosis [11, 18, 33], renal failure [13, 17, 20], and sepsis [18]. This may be due to abnormalities that occurred in the patients.

In this review, investigations found that all cases had genitourinary anomalies [1–3, 5–7, 9–13, 15–17, 19, 22, 23, 25–27, 29–34], with the most common anomaly was fistulas. The majority of the fistula were urethrorectal fistulas [1, 13, 16, 17, 19, 22, 23, 25, 27, 29–31, 33, 34] followed by urethroperineal fistula [2, 7, 20, 25]. The majority of abnormalities after fistula in the genitourinary system occurred in the kidneys. Hydronephrosis is the most common anomaly in kidneys [2, 8–11, 13, 18, 19, 25, 30, 31, 33, 34], followed by renal dysplasia [2, 12, 13, 17, 20, 23, 24, 33], and renal agenesis [11, 14, 15, 25, 30]. In the ureter, most anomalies were vesicoureteral reflux [2, 9, 10, 13, 15, 19, 22, 28, 33]. There were also found a small number of anomalies such as hypoplastic renal [3, 5], duplication of renal [2], absence of ureter [2, 15, 20], ectopic ureter [2], ureterocele [15], absence of bladder [2, 12, 20], absent [15] or hypoplastic prostate [23], and absence of urethra [3, 24, 26, 32].

Apart from abnormalities in the genitourinary system, in the respiratory system [1, 11, 14, 24], cardiovascular system [1, 13, 14, 24], digestive system [21, 26, 32], and musculoskeletal system abnormalities were also found in a small number of cases [13, 24].

The management of each case can be different because almost every case had different anomalies. Cystostomy was performed in most cases as the initial management of aphallia [7–11, 13, 16, 18, 20–22, 26, 27, 32, 33], especially those found to have a fistula, to allow draining of the urine, and to correct the fistula.

For advanced management, phalloplasty was more often done compared to patients who choose the female sex. Although aphallia is associated with congenital abnormalities, the mortality rate is quite low, and is caused by complications mainly due to sepsis and uricemia [13, 21, 25, 31].

Aphallia management is divided into three distinct phases: acute, subacute, and chronic. Previously, aphallia patients needed to be checked for chromosomes to exclude other causes of sexual differentiation and abnormalities such as congenital adrenal hyperplasia or other penile abnormalities (severe hypospadias, epispadias, and hidden or micropenis) [35].

In the acute phase, therapy is supportive and involves managing life-threatening complications. Vesicostomy can be performed, especially in patients who present with sepsis or urinary tract obstruction due to the absence of the urethra, to drain the urinary tract, relieve pressure, and prevent infection. In aphallia, urine obstruction is not always present, as seen in the case of urethra-perineal fistula, and no urine transfer is required at this stage [35].

Subacutely, perineal urethrostomy or catheterization vesicostomy can be performed for the separation of urethrorectal fistulas and in patients with a urethra that is too small or absent in order to gain access to the bladder [16]. Additionally, a pseudophallus can be created to enable patients to identify themselves as male [16].

The gender determination of aphallia is still controversial. In the past, surgery was recommended as the only reconstructive option in these cases, known as feminization genitoplasty. This recommendation was based on the belief that raising these patients as males could be disruptive, and it was considered better to be an imperfect female than an inadequate male. The management strategy includes early bilateral orchidectomy to prevent postnatal androgen implantation, followed by vaginal interposition and hormonal therapy during puberty. However, these patients often experience high levels of gender dysphoria, due to prenatal androgen implantation [35].

Prenatal and early neonatal androgen imprinting has been studied in animal models, pregnant women undergoing hormone therapy, and children with congenital adrenal hyperplasia. It has been shown that fetal exposure to testosterone has an influence on childhood sex interests, such as playmates and activity preferences, as well as later sexual orientation, which tends towards typically male behaviors and gynecophilia [35].

Sex determination considerations should be based on the sex that provides the best prognosis in terms of reproductive function, the ability to function sexually, the appearance of the external genitalia, and the patient’s self-identification with a particular sex. Therefore, although some authors still recommend feminizing genitoplasty for infants and newborns, it is now generally accepted that genetically normal males with aphallia should be supported surgically as males until the patient is old enough to identify their own sex, thus reducing gender dysphoria [35].

Chronic management is to create the functional prosthetic and aesthetic neophallus [16]. Construction of a functional neophallus requires a multidisciplinary team approach. One of the most considered treatment options for long-term management is phalloplasty, which can be performed before the patient reaches puberty. The gold standard technique for phalloplasty is microsurgical phalloplasty with a radial forearm free flap, which has shown good results and positive outcomes in terms of patients’ self-esteem and sexual well-being [36].

This study has several limitations. This review includes only studies published in English and may result in publication bias (language) in selecting studies due to the exclusion of other relevant articles published in languages other than English. Some articles lack data regarding patients, including age, clinical presentation, investigations, and evaluation of family history. This may be due to the rarity of cases and the variability of cases of aphallia, so that there is no standard method of aphallia. In addition, evaluating the progression of aphallia treatment is a challenge because many studies have not reported the long-term progress of the treatment chosen. Long-term follow-up is essential, but there is a distinct lack of information regarding the long-term outcomes of these patients. These patients need lifelong follow-up in a team of specialists, especially after puberty.

## Conclusions

This systematic review shows that aphallia is a rare congenital anomaly, and all cases were associated with other genitourinary congenital abnormalities, especially fistulas between the genital tract and the rectum. Although often associated with other congenital abnormalities, most physical examinations and laboratory tests showed normal results except for the phallus. Initial management generally includes vesicostomy, and for advanced management, sex determination is recommended. Currently, male sex is preferred over female sex, possibly due to the predominance of male patients.

## Data Availability

All data generated or analysed during this study are included in this published article.
